# Meso-scale network analysis of resting state-fMRI brain network connectivity performs poorly as a prognostic tool in critically ill traumatic brain injury patients

**DOI:** 10.1016/j.ynirp.2022.100079

**Published:** 2022-01-10

**Authors:** Jonathan Tjerkaski, William H. Thompson, Bo-Michael Bellander, Eric P. Thelin, Peter Fransson

**Affiliations:** a Department of Clinical Neuroscience, Karolinska Institutet, Stockholm, Sweden; b Department of Neurosurgery, Karolinska Institutet, Stockholm, Sweden; c Department of Neurology, Karolinska University Hospital, Stockholm, Sweden

**Keywords:** Functional magnetic resonance imaging, Traumatic brain injury, Graph theory, Prognostication

## Abstract

Prognostication is important in the management of critically ill traumatic brain injury (TBI) patients, as it may guide decisions on treatment intensity. Changes in functional connectivity that arise due to TBI have been observed in several brain networks using resting-state functional magnetic resonance imaging (rs-fMRI). Studies suggest that cognitive dysfunction following TBI can, in part, be explained by changes in functional connectivity. The overall aim of this study was to investigate whether easy-to-calculate graph theory-based summaries of brain network functional connectivity can discriminate between patients with favorable and unfavorable outcomes. To further substantiate these results, we used the Louvain algorithm to detect the community structure of each TBI patient, to assess whether it differs from that of healthy individuals.

We performed fMRI on 44 TBI patients who were admitted to the neurocritical care unit. Long-term functional outcome was determined at 6–12 months using the Glasgow outcome scale (GOS), with GOS 1–3 being defined as an unfavorable outcome. Functional connectivity was measured using Pearson’s correlation coefficient. We derived topographical summaries of brain network connectivity using graph theory. We computed the participation coefficient (PC) and the module degree z-score (MDZ) for each parcel in the cortical template of Thomas Yeo et al. (2011). As topographical summaries of the network parameters we used the median, maximum and standard deviation (SD) of PC and MDZ in each of the seven functional networks that are included in the cortical parcellation of Yeo et al. Age and sedative medications were used as nuisance regressors.

Using multivariate logistic regression, we observed no statistically significant association between the topographical summaries of brain connectivity and the dichotomized GOS. Thus, although graph theoretical measures applied to brain network connectivity patterns have previously been reported to be associated with cognitive dysfunction, they appear to be unable to predict long-term functional outcomes in critically ill TBI patients. Similarly, the extent to which TBI patient’s community structures differed from that of healthy individuals, measured using adjusted mutual information, was also not associated with the dichotomized GOS.

In summary, our results suggest that Meso-scale graph theoretical analyses of rs-fMRI brain network connectivity patterns can be expected to perform poorly as a prognostic tool in critically ill TBI patients.

## 1 Introduction

Traumatic brain injury (TBI) is the most common cause of death in young adults (Jennett, 1996), and survivors may suffer from lifelong disabilities (Draper et al., 2007). Prognostication is important in the management of critically ill TBI patients, as it may guide decisions on treatment intensity. Biomarkers that can accurately predict functional outcomes after TBI are thus of great interest. Such biomarkers would also play an instrumental role in facilitating the design of individualized treatment plans in the neuro-rehabilitation clinic, to improve recovery and long-term cognitive performance.

There is considerable evidence that suggests that resting-state functional connectivity is affected in traumatic brain injury (TBI) (Ham and Sharp, 2012). Studies in TBI patients have revealed widespread alterations in functional connectivity relative to healthy controls. Changes in functional connectivity that arise due to TBI have been observed in networks such as the default mode network (DMN) (Sharp et al., 2011), salience network and frontoparietal networks (Hillary et al., 2011; Bonnelle et al., 2012), as well as motor networks (Kasahara et al., 2010). Studies suggest that such changes in functional connectivity may be associated with impairments in **consciousness,** memory, processing speed and attention, in addition to other important cognitive functions (Sharp et al., 2011; Moore et al., 2020; Shumskaya et al., 2017; Kondziella et al., 2017; Threlkeld et al., 2018). Furthermore, graph theoretical approaches applied to brain network connectivity patterns in TBI patients have shown that key brain networks involved in supporting cognitive function exhibit decreased “small-worldness” in their organization (Pandit et al., 2013).

The overall aim of this study was to investigate whether graph theoretical approaches applied to resting-state functional magnetic resonance imaging (rs-fMRI) brain network connectivity patterns can discriminate between TBI patients with favorable and unfavorable outcomes, thereby contributing to prognostication in TBI. We hypothesized that topographical summaries of the default mode network will show decreased participation coefficient (PC) and increased module degree z-score (MDZ) and that these measures would be associated with long-term functional outcomes in critically ill TBI patients. These measures were chosen as they allow for the quantification of meso-scale changes both within and between networks, presenting a possible easy-to-calculate network “biomarker” for TBI severity by detecting alterations in the functional connectivity of brain networks.

Additionally, we examined the association between the inter-hemispheric connectivity and traumatic axonal injuries (TAI) in the corpus callosum, in order to determine whether changes in functional connectivity could be attributed to a specific focal injury such as TAI.

## 2 Materials and methods

The methodology of this study was registered prior to the commencement of data analysis (link to pre-registration: https://osf.io/bzh2g).

### 2.1 Study design

This was a prospective observational study. The study protocol was approved by the Stockholm County ethical committee (#2010/1072-31/1). All included study participants were blunt TBI patients, aged 15 or older, who were admitted to the neurocritical care unit (NCCU) at Karolinska University Hospital following TBI, according to the clinical assessment of the attending neurosurgeon. A complete rs-fMRI examination was performed as soon as the medical condition of each study participant was considered sufficiently stable for transportation to the radiology department. Patients were included based on the availability of author BMB.

Patients with previous TBI, spontaneous intracerebral hemorrhage, aneurysmal subarachnoid hemorrhage, ischemic stroke, acute bacterial meningitis, or other serious pathology of the brain were excluded. Furthermore, study participants were excluded if the manual evaluation of the images and/or the results of pre-processing revealed that the quality of the images and/or the result of their processing during image analysis was inadequate. Long-term functional outcome was determined at 6–12 months using the Glasgow outcome scale (GOS) (Jennett and Bond, 1975). GOS is routinely assessed at our institution during follow-up appointments and/or by structured questionnaires. The latest available GOS was used. The primary outcome measure of this study was a dichotomized GOS, where we defined unfavorable outcome as GOS 1–3 and favorable outcome as GOS 4–5. We also documented the administration of sedative medication to each study participant during the 24 h that preceded their MRI examination.

### 2.2 Treatment

Treatment at the NCCU at the Karolinska University Hospital adheres to the guidelines set forth by the Brain Trauma Foundation (Guidelines for the Manage, 2007). Patients with an impaired level of consciousness following TBI were intubated, mechanically ventilated, and sedated. Intracranial mass lesions were evacuated as deemed appropriate by the attending neurosurgeon. Intracranial pressure (ICP) and mean arterial pressure (MAP) were measured invasively. Cerebral perfusion pressure (CPP) was calculated as MAP-ICP. Patients' heads were elevated at an angle of 30 degrees. ICP was targeted at ≤20 mm Hg, CPP was targeted at 50–70 mm Hg. Treatment targets were achieved with the use of intravascular infusions, vasopressors, osmotic therapy, intermittent cerebrospinal fluid (CSF) drainage, mild hyperventilation, mild temperature control (35–36 °C), barbiturate coma and decompressive craniotomy as needed. Patients were transported to the radiology department for rs-fMRI at a time when their ICP was deemed to be stable by the treating physician. The study participants were sedated at the time of the rs-fMRI examination using continuous infusions of clonidine, propofol, midazolam, opioids or combinations thereof, at the discretion of the treating physician.

### 2.3 MRI scanning protocol

The conventional MRI protocol included Susceptibility-weighted imaging (SWI, 3D, TR/TE = 49/40 ms, flip = 15 deg, FOV = 217 × 240mm, slice thickness = 1.6 mm), fluid-attenuated inversion recovery (FLAIR, 2D, TR/TE = 9000/119 ms, inversion time = 2500 ms, flip = 150 deg, FOV = 191 × 240 mm) and diffusion-weighted imaging (DWI, TR/TE = 4300/94 ms, flip = 90 deg, FOV = 240 × 240 mm, b = 3000, six directions). Additionally, rs-fMRI data was acquired using a T2*‐weighted gradient-recalled echo-planar imaging (EPI) sequence with the following protocol: TR = 2500 ms, TE = 40 ms, flip angle = 90 degrees, voxel size = 2 × 2x2 mm^3^, number of image volumes = 210 (including dummy scans).

### 2.4 rs-fMRI data analysis

#### 2.4.1 Data acquisition and pre-processing

In each patient, 8 min 30 s (510 s) of rs-fMRI data was recorded. The first four scans (10 s) were not used in the analysis, leaving 500 s worth of data per patient. Image pre-processing and resting-state fMRI connectivity analysis was performed using the default functional pipeline of the SPM12 software platform (Wellcome Center for Neuroimaging, London) and the CONN toolbox v19.c (Whitfield-Gabrieli and Nieto-Castanon, 2012) in Matlab (Mathworks Inc., MA, USA). Pre-processing steps included denoising, slice timing correction, co-registration and spatial normalization. Spatial smoothing was not used. White-matter and cerebrospinal fluid confounds were calculated using the component-based noise correction method (CompCor (Behzadi et al., 2007)). Data denoising steps also included linear de-trending and band-pass filtering (0.008–0.1 Hz).

#### 2.4.2 Managing signal dropout and brain parcellation

Due to the presence of artifacts that arise either as a consequence of trauma or due to surgical interventions, we suspected that there would be voxels with an abnormal signal intensity that is not representative of the BOLD-signal. We followed an identical procedure to Thompson et al. (2021) by removing voxels from being included in a parcel based on the distribution of average voxel intensity (Thompson et al., 2021). This procedure entailed identifying “good voxels” and “bad voxels”. To achieve this, we used Gaussian Mixed Models (GMM) which were applied to the average voxel intensity over time of all voxels within the gray matter masks of each study participant. This was run for k = 1 to k = 5 clusters (i.e., number of Gaussians to model the distribution). After a manual inspection of the clustering, we chose a value of k = 4. With this value for k, we removed a lower-bound tail for all subjects (if present). By doing so, we excluded all voxels that were classified into the Gaussian with the smallest mean.

The pre-processed data was then segmented into 400 parcels using the Schaefer et al. (2018) parcellation (Schaefer et al., 2018) using only the “good voxels” identified from the previous step. If more than 50 percent of a parcel consisted of “bad voxels”, the entire parcel was left out from the network analysis.

Additionally, we considered that the excluded voxels (“Bad voxels”) for each study participant could potentially prove to be a parsimonious biomarker in TBI, as injuries such as cerebral contusions may lead to BOLD signal loss. Therefore, we also examined the relationship between the percentage of excluded voxels and the long-term functional outcome in TBI patients.

#### 2.4.3 Network theory measures

Connectivity was estimated using Pearson correlations. The participation coefficient (PC) and module degree z-score (MDZ) were calculated using the Yeo 7 (2011) template as a community vector (Thomas Yeo et al., 2011; Guimerà and Amaral, 2005). Only positive connections were considered when estimating PC values. Next, given the heterogeneous nature of TBI, we aimed to create estimates that generalize the connectivity profiles of each brain network. Thus, from the PC and MDZ estimates, which calculate a value for each node, we derived topographical summaries of the network parameters. We used the median, maximum and standard deviation (SD) of PC and MDZ, respectively, of each network.

#### 2.4.4 Quantifying the derived community partition deviation from the community template

In addition to examining topographical summaries, we also wanted to investigate whether the communities of patients differed from a standardized template and whether this difference corresponded to the severity of the disorder. The standardized cortical template of Yeo et al. was developed in primarily healthy human subjects. It is plausible that the extent to which a patient differs from the template corresponds to the severity of the TBI. Therefore, we first estimated communities for each subject using the Louvain algorithm on positive edges. This was done using the python version of the brain connectivity toolbox (bctpy, version 0.5.0). The resolution parameter was fixed to 1 for all subjects. We then calculated the adjusted mutual information (AMI) to examine the overlap between the cortical template of Yeo et al. and the derived community partition for each TBI patient. We subsequently examined whether the degree to which TBI patient’s community structures deviate from the norm was predictive of long-term functional outcomes.

### 2.5 Structural MRI

TAI was documented in the corpus callosum according to the protocol of Tjerkaski et al. (2021). In brief, TAI was defined as hypo-intensities on susceptibility-weighted imaging (SWI), an increase in signal intensity on fluid-attenuated inversion recovery (FLAIR) images and restricted diffusion on diffusion-weighted imaging (DWI), with examples provided in the supplementary materials (Supplemental Figs. 1–3). Restricted diffusion on DWI was, in turn, defined as a hyper-intensity on the isotropic diffusion map with a simultaneous hypo-intensity on the Apparent diffusion coefficient (ADC)-map, in the same anatomical location. TAI was marked as either “present” or “absent” in the corpus callosum, using any of the above-mentioned MRI pulse sequences. Iatrogenic injuries, such as those caused by the insertion of external ventricular drains, were disregarded.

### 2.6 Statistical analysis

Groupwise comparisons were carried out between the group of TBI patients who had an unfavorable outcome and those who had a favorable outcome. Differences concerning continuous and categorical variables were assessed using the Mann–Whitney *U* test and the Chi-square test, respectively.

The relationship between the dichotomized GOS and connectivity measures was examined using multivariate logistic regression. Age and the administration of sedative medications (Propofol, Midazolam, Clonidine and Opioid analgesics) were used as nuisance regressors. The predictors were thus the median, maximum and standard deviation (SD) of the PC and MDZ values, respectively, for the DMN, as well as the nuisance regressors. Although not stated in the pre-registration, we subsequently repeated the statistical analysis in all seven of the functional networks that are included in the Yeo 7 2011 template (Thomas Yeo et al., 2011), as opposed to only investigating the DMN. In order to examine whether the exclusion of “bad voxels” may have introduced a degree of bias to our results, we have fitted additional logistic regression models which include the proportion of excluded voxels as a nuisance regressor.

Multi-collinearity between the predictors was assessed prior to modeling. In the case of multicollinearity (Pearson’s correlation coefficient exceeding the absolute value of 0.3), the included predictors were prioritized in accordance with the protocol given in the pre-registration. In brief, the median PC and MDZ values were prioritized ahead of the SD and maximum values. Similarly, the summaries of the PC values were prioritized ahead of the summaries of the MDZ values, for each functional network.

We used logistic regression to examine the association between inter-hemispheric connectivity and TAI in the corpus callosum, with the presence of TAI in the corpus callosum as the dependent variable and both inter-hemispheric connectivity and the nuisance regressors as the independent variables. We assessed both the median of the global inter-hemispheric connectivity and the median inter-hemispheric connectivity specifically in DMN structures, respectively.

The relationship between functional outcome and the percentage of excluded parcels, as well as the relationship between functional outcome and AMI, were also examined using logistic regression, like that which was mentioned above concerning the analysis of connectivity measures and inter-hemispheric connectivity.

Model performance was assessed using Nagelkerke’s Pseudo-R^2^, a measure of goodness-of-fit, and the area under the receiver operating characteristic curve (AUROC), a measure of discrimination. Additionally, we estimated Akaike’s information criterion (AIC), to facilitate the comparison of models that have an unequal number of predictors. Moreover, we used the likelihood ratio test in order to further examine the differences between the logistic regression model which only consisted of the nuisance regressors and those which also contained connectivity measures.

### 2.7 Levels of statistical significance and multiple comparison correction

False discovery rate (FDR) was used to correct for multiple statistical tests within hypotheses. All hypotheses are considered by different statistical families as they are asking different research questions regarding fMRI activity (functional connectivity, bilaterality, AMI and the proportion of excluded voxels due to poor BOLD-signal). Statistical significance was set at p = 0.01.

## 3 Results

### 3.1 Demographics

Approximately half (n = 21) of the included study participants (n = 44) had an unfavorable long-term functional outcome (Table 1). Patients who had an unfavorable outcome were, on average, older than those who had a favorable outcome (Mann-Whitney *U* test, p = 0.02). MRI examinations were performed at a median of 8 and 7 days in patients with favorable and unfavorable outcomes, respectively. The majority of study participants were classified as having suffered severe TBI (80%), while only a single study participant had mild TBI. We did not record any deaths due to withdrawal of life sustaining therapy at the ICU. However, two study participants died after discharge from the ICU, for whom cause of death is unknown.

**Table 1:** Demographics.

	favorable (N = 23)	unfavorable (N = 21)	p value
**Age (years)**			0.016^1^
Median (Q1, Q3)	33.0 (22.5, 56.5)	60.0 (41.0, 68.0)	
Range (Min - Max)	16.0–68.0	19.0–73.0	
**GCS**			0.257^1^
Median (Q1, Q3)	6.0 (3.0, 9.5)	4.0 (3.0, 6.0)	
Range (Min - Max)	3.0–14.0	3.0–11.0	
**Pupillary response**			0.135^2^
Responsive	20 (87.0%)	13 (61.9%)	
Unilaterally unresponsive	3 (13.0%)	7 (33.3%)	
Bilaterally unresponsive	0 (0.0%)	1 (4.8%)	
Propofol administration*			0.020^2^
Yes	21 (91.3%)	13 (61.9%)	
No	2 (8.7%)	8 (38.1%)	
Midazolam administration*			0.242^2^
No	15 (65.2%)	17 (81.0%)	
Yes	8 (34.8%)	4 (19.0%)	
Opioid analgesia administration*			0.392^2^
No	16 (69.6%)	12 (57.1%)	
Yes	7 (30.4%)	9 (42.9%)	
Clonidine administration*			0.252^2^
No	22 (95.7%)	18 (85.7%)	
Yes	1 (4.3%)	3 (14.3%)	
**NCCU stay duration (days)**			0.511^1^
Median (Q1, Q3)	14.6 (9.8, 19.3)	16.0 (11.7, 21.8)	
Range (Min - Max)	5.5–30.7	6.0–29.3	
**Time until the MRI examination (days from trauma)**			0.723^1^
Median (Q1, Q3)	7.5 (5.0, 12.8)	7.0 (5.0, 13.0)	
Range (Min - Max)	1.0–23.0	1.0–27.0	
**TBI severity**			0.487^2^
Severe (GCS 3–8)	17 (73.9%)	18 (85.7%)	
Moderate (GCS 9–12)	4 (17.4%)	3 (14.3%)	
Mild (GCS 13–15)	2 (8.7%)	0 (0.0%)	
**Time until outcome assessment†**			0.305^1^
Median (Q1, Q3)	361.0 (335.5, 382.5)	357.0 (317.0, 363.0)	
Range (Min - Max)	93.0–450.0	176.0–398.0	

1: Mann-Whitney *U* test, 2: Pearson’s Chi-squared test, *: At the time of magnetic resonance imaging. † Excluding patients with GOS 1 (n = 3), as the outcomes of these patients were assessed at ICU discharge or shortly thereafter. abbreviations: NCCU = Neurocritical care unit, MRI = Magnetic resonance imaging, Q1 = First quartile, Q3 = Third quartile.

### 3.2 Topographical summaries

The values for the topographical summaries of functional connectivity that were used in the main analysis of this study are presented in Table 2, alongside the AMI values and the percentage of excluded voxels. We observed statistically significant differences in the median PC value of the Limbic network and median MDZ of the control network, respectively, when comparing patients with favorable and unfavorable outcomes (Mann-Whitney *U* test, p = 0.042 and p = 0.048). Although, this comparison does not consider the nuisance regressors and these results must therefore be interpreted with caution.

**Table 2:** Topographical summaries of brain network functional connectivity.

Metric	Network	Measurement	favorable (N = 23)	unfavorable (N = 21)	p value*
**Median Participation Coefficient**	Control	Median (Q1, Q3)	0.810 (0.783, 0.820)	0.813 (0.794, 0.827)	0.272
	DMN	Median (Q1, Q3)	0.781 (0.729, 0.804)	0.791 (0.760, 0.816)	0.175
	DorsAttn	Median (Q1, Q3)	0.820 (0.802, 0.827)	0.825 (0.809, 0.831)	0.339
	Limbic	Median (Q1, Q3)	0.819 (0.814, 0.836)	0.829 (0.825, 0.836)	0.042
	SalVentAttn	Median (Q1, Q3)	0.811 (0.789, 0.823)	0.820 (0.807, 0.827)	0.116
	Somatomotor	Median (Q1, Q3)	0.762 (0.738, 0.813)	0.802 (0.748, 0.815)	0.592
	Visual	Median (Q1, Q3)	0.803 (0.783, 0.818)	0.806 (0.787, 0.822)	0.852
**Median Module degree Z-score**	Control	Median (Q1, Q3)	0.153 (0.017, 0.206)	−0.005 (−0.046, 0.125)	0.048
	Limbic	Median (Q1, Q3)	0.082 (−0.009, 0.214)	0.106 (−0.001, 0.288)	0.339
	SaltVentAttn	Median (Q1, Q3)	0.051 (−0.018, 0.161)	0.034 (−0.078, 0.054)	0.116
**Other**	AMI	Median (Q1, Q3)	0.191 (0.127, 0.244)	0.167 (0.071, 0.208)	0.253
	Excluded voxels	Median (Q1, Q3)	12.070 (10.769, 14.436)	13.657 (12.907, 15.221)	0.056

* Mann-Whitney *U* test. Abbreviations: Q1 = First quartile, Q3 = Third quartile.

### 3.3 Outcome prediction

Due to the correlations between the median MDZ and the median PC values for the somatomotor, DorsAttn, DMN and visual networks exceeding a Pearson’s correlation coefficient of 0.3, we only included the median PC values of these functional networks in subsequent analyses. As the correlations between the median PC and median MDZ values of the Limbic, SalVentAttn and Control networks were below an absolute value of 0.3, we used both the median PC and the median MDZ when examining the association between these topographical summaries of resting state functional connectivity for these brain networks and long-term functional outcome in TBI patients. However, we omitted the maximum and SD of the PC and MDZ values from subsequent analyses for all functional networks as their correlations with the median PC and/or median MDZ values for the same brain network exceeded a Pearson’s correlation coefficient of 0.3.

The predictive performance of the investigated connectivity measures is shown in Table 3, Table 4. In general, we found that the performance of multivariate logistic regression models that included variables that were derived from the rs-fMRI analysis was not significantly different from those models which solely consisted of the nuisance regressors. This was confirmed using the likelihood ratio test, which did not reveal any statistically significant differences between the model which only consisted of the nuisance regressors and those that contained connectivity measures (p > 0.05).

**Table 3:** Overall performance of logistic regression models.

Model	Pseudo-R^2^	AUROC	AIC
Nuisance regressors	0.36	0.80	58.9
DMN + Nuisance regressors	0.39	0.81	59.7
Somatomotor + Nuisance regressors	0.37	0.81	60.8
Visual + Nuisance regressors	0.37	0.81	60.8
SalVentAttn + Nuisance regressors	0.48	0.85	57.5
DorsAttn + Nuisance regressors	0.39	0.83	59.8
Control + Nuisance regressors	0.46	0.82	58.3
Limbic + Nuisance regressors	0.41	0.83	60.6
Excluded voxels + Nuisance regressors	0.38	0.80	60.3
AMI + Nuisance regressors	0.48	0.82	55.5

**Table 4:** Coefficients and p-values for each of the investigated connectivity measures.

Model	Predictor^a^	Coefficient	p-value^2^	Adj p-value
SalVentAttn + Nuisance regressors	Median MDZ	−10.42	0.06	0.31
Control + Nuisance regressors	Median MDZ	−6.04	0.06	0.31
Limbic + Nuisance regressors	Median PC	47.02	0.22	0.52
SalVentAttn + Nuisance regressors	Median PC	25.59	0.29	0.52
DMN + Nuisance regressors	Median PC	10.13	0.30	0.52
DorsAttn + Nuisance regressors	Median PC	24.03	0.31	0.52
Excluded voxels + Nuisance regressors	Excluded voxels (%)	7.50	0.48	0.48
Limbic + Nuisance regressors	Median MDZ	1.24	0.60	0.79
AMI + Nuisance regressors	AMI	−2.00	0.64	0.64
Visual + Nuisance regressors	Median PC	−4.77	0.75	0.79
Control + Nuisance regressors	Median PC	5.08	0.77	0.79
Somatomotor + Nuisance regressors	Median PC	2.36	0.79	0.79

a : All of the included predictors are shown in this column, all other predictors were discarded due to multicollinearity, as discussed in the methodology section. 2: p values were estimated using the Wald test.

We used AMI to estimate the degree of overlap between the community structure that was empirically detected in our cohort of TBI patients, using the Louvain algorithm, and the standardized cortical template of Yeo et al. We did not observe any statistically significant association between AMI and long-term functional outcome (Table 3, Table 4). Likewise, we did not observe any association between the number of excluded voxels and long-term functional outcomes. No significant differences to the results presented in Table 3, Table 4 were observed when adding the proportion of excluded voxels to the nuisance regressors (Supplementary materials, Supplemental Tables 1 and 2).

In summary, we concluded that none of the investigated measures that were derived from rs-fMRI had any statistically significant relationship to the dichotomized GOS after adjusting for nuisance regressors in our patient cohort (Table 4).

### 3.4 Inter-hemispheric connectivity

We observed no statistically significant relationship between TAI in the corpus callosum and inter-hemispheric connectivity in structures belonging to the DMN (p = 0.92) and inter-hemispheric connectivity of the whole brain (0.78), respectively.

## 4 Discussion

In this study, we hypothesized that there would be a statistically significant association between connectivity measures derived using rs-fMRI and long-term functional outcomes in critically ill TBI patients. This hypothesis was based on a sound body of evidence, suggesting that connectivity measures are associated with alternative outcome measures of TBI, such as cognitive function testing (Sharp et al., 2011; Moore et al., 2020; Shumskaya et al., 2017). Nevertheless, we were unable to detect any statistically significant relationships between meso-scale graph theoretical measures applied to brain network connectivity patterns and long-term functional outcome in this cohort of critically ill TBI patients. The degree to which the community structure of TBI patients deviates from that of healthy controls, assessed using AMI, was not predictive of long-term functional outcomes. In summary, our findings suggest that PC and MDZ values are not significantly associated with functional outcome in critically ill TBI patients.

Although it is possible to also calculate negative correlations and thereby obtain negative edges in our network model, we decided to limit our analysis to positive correlations for two reasons. First, and most importantly, there is an on-going debate of how negative correlations are to be interpreted in a neurobiological setting. Moreover, negative correlations need to be interpreted with great care since specific steps in the data pre-processing pipeline (i.e. global signal regression, GSR) might affect the strength of negative correlations (K and MD, 2017). Additionally, the conventional definition of the participation coefficient network parameter used here only handles positive edges.

We used GMM to classify voxels according to their signal intensity over time. A lower-bound tail corresponding to voxels with negligible signal intensity was, if present, excluded from further analysis. The proportion of excluded voxels, also referred to as “bad voxels”, did not have any statistically significant association to functional outcome. Therefore, while we were unable to confirm the hypothesis that the proportion of bad voxels correlates with clinical outcome, we also believe that it is unlikely that the removal of bad voxels systemically biases the subsequent analyses.

The use of rs-fMRI can be considered somewhat precarious in critically ill TBI patients, due to the pathological changes that may occur in these patients, as well as the medical treatment that they receive (e.g., medically induced coma) (Wang et al., 2020; Purdon et al., 2009; Vatansever et al., 2020). In particular, the presence of intracranial hemorrhage, intracranial air and intracranial monitoring devices could potentially interfere with the T2*‐weighted gradient-recalled echo-planar imaging (EPI) sequences that are commonly used in conjunction with rs-fMRI analysis (Cordes et al., 2000), resulting in both distortions and signal dropout due to magnetic field inhomogeneity. Although we excluded voxels that had abnormal signal loss using GMM, we nevertheless suspect a certain degree of interference due to image artifacts. Thus, we believe that there is a need for novel pre-processing tools that are specifically tailored to image analysis in NCCU patients. Moreover, certain alternative methods of fMRI data acquisition, such as arterial spin labeling (Petersen et al., 2021) and off-set spin-echo EPI (Barghoorn et al., 2021), could perhaps mitigate interference due to changes in magnetic susceptibility, albeit at the expense of temporal resolution (Jann et al., 2015).

In this study, we observed statistically significant differences in the median PC value of the Limbic network and the median MDZ of the control network when comparing patients with favorable and unfavorable outcomes using the Mann-Whitney *U* test. However, no such differences were observed when adjusting for the nuisance regressors in logistic regression models. These groupwise differences in the topographical summaries of functional connectivity patterns likely reflect differences in age or the administration of sedative medications, rather than the pathology of TBI.

Standardized cortical templates are generally derived from data that was obtained from healthy individuals, as opposed to severely brain injured patients. We speculated that there could be a discrepancy in the community structure of critically ill TBI patients and the parcellation of Yeo et al., which we used when estimating brain network connectivity patterns. Moreover, we believed that the degree to which the functional architecture of TBI patients deviates from that of healthy individuals might be related to neurological outcomes. Therefore, we computed AMI to assess of the overlap between the network parcellation of Yeo et al. and the community structure of each TBI patient. Nevertheless, we did not observe any statistically significant association between AMI and outcomes in TBI patients. These results provide further evidence for the notion that resting-state functional connectivity is of limited prognostic value in critically ill TBI patients.

In contrast to previous studies, we were unable to detect any significant relationship between TAI and functional connectivity measures (Jolly et al., 2020). However, previous studies that have investigated the relationship between injuries in white matter tracts and connectivity measures derived using rs-fMRI have done so using measurements of fractional anisotropy, rather than radiological assessments of TAI detected using conventional MRI (e.g. (Jolly et al., 2020)). The sensitivity of conventional MRI to axonal pathology has been shown to be inferior to that of diffusion-tensor imaging (DTI) (Jolly et al., 2021). Thus, the lack of an association between functional connectivity measures and TAI that was observed in this study may be confounded by the poor sensitivity of conventional MRI. Nevertheless, it has also been reported that focal lesions in TBI only weakly influence the ability of functional network hubs to coordinate processing in brain networks, which could potentially explain the lack of an association between inter-hemispheric connectivity and TAI in the corpus callosum that was observed in this study (Reber et al., 2021).

Although graph theoretical measures applied to brain network connectivity patterns are correlated to alternative outcome measures in TBI (Cao et al., 2021; Grossner et al., 2019), this does not appear to be the case for GOS. It is also possible that other global network measures, such as the average path length or network efficiency might provide answers that the PC and the MDZ cannot. Nevertheless, we elected to limit the number of measurements used to decrease the potential risk of type 1 error. The discrepancy between our results and those of previous studies can likely be attributed to differences in outcome measures. While generally correlated to both neuropsychological testing and measures of quality of life (Wilson et al., 2000), GOS has also been shown to be only weakly associated with certain measures of cognitive function (Zou et al., 2020). More granular testing of cognitive function would presumably be necessary to corroborate the findings previously described in the literature pertaining to the association between brain connectivity and outcomes in TBI patients. A limitation of this study is thus that data on cognitive function was not obtained as part of the study protocol. An additional source of discrepancies is the fact that other forms of fMRI analysis, such as seed-based methods, were also not investigated in the present study. Although we consider this beyond the scope of the present study, we do encourage future investigations with the purpose of examining whether other modalities for fMRI analysis, such as seed-based methods, might prove more effective and robust than graph theoretical measures in discriminating between TBI patients with favorable and unfavorable outcomes.

Further, another limiting aspect was that our graph theoretical approach aimed at identifying stereotypical network changes in a cohort of patients that suffer from a highly heterogeneous medical condition. Thus, the combination of a highly heterogeneous study population, high-level summaries of network measures and a relatively rough outcome measure (GOS) may simply be too imprecise to consistently identify group-level differences between TBI patients that have favorable and unfavorable neurological outcomes. For instance, we did observe trends suggesting that the median MDZ values for the SalVentAttn and Control networks might be used to distinguish patients with favorable and unfavorable outcomes, although these trends did not quite reach statistical significance. It is entirely possible that if the data were less heterogeneous and if the sample size was greater that some of the analyses presented in this paper, such as those concerning the median MDZ value for the SalVentAttn and Control networks, would become statistically significant. While that is a possibility, it is not something we can address in this present study. However, we must also emphasize that lengthy MRI examinations are not without risk in critically ill TBI patients. We strongly believe that any and all interventions that are carried out in such a patient population must, beyond any reasonable doubt, be of a net benefit to the patient. This is essentially why a more stringent significance level of p = 0.01 was chosen for the present study, as opposed to the more conventional threshold of 0.05. We are not convinced that meso-scale network analysis of resting state-fMRI brain network connectivity using PC and MDZ meet this relatively high burden of proof. Nevertheless, we do believe that more targeted network analysis approaches, combined with cognitive-specific outcome measures, may be an appropriate alternative in future analyses.

A major difference between this study and many other studies is that we specifically set out to compare critically ill TBI patients who had unfavorable outcomes with critically ill TBI patients who had favorable outcomes, as opposed to comparing TBI patients with healthy controls. We believe that the differences between two groups of critically ill TBI patients probably are much smaller than the differences between TBI patients and healthy controls, regardless of the outcome measures or analysis methods used. Although, this hypothesis will have to be confirmed in future scientific studies.

A limitation of this study is that we did not document the study participant’s levels of sedation prospectively. Therefore, we could not account for the level of sedation in our statistical models. One might speculate that patients with a favorable outcome were sedated to a greater degree at the time of rs-fMRI than patients with unfavorable outcomes, as patients with favorable outcomes may be awake and agitated in the sub-acute setting following TBI. However, it is uncertain whether differences in drug administration influenced the results of this study, given that we did adjust for the administration of several medications in our statistical models. Furthermore, the changes in functional connectivity that are associated sedative medication have been shown to be relatively small compared to those that originate from structural brain injury (Kirsch et al., 2017).

In this study, we specifically chose to examine PC and MDZ because they offer an intuitive summary of brain network-levels of integration (i.e. between brain network communication) and segregation (i.e. within brain network communication). Previous studies have successfully utilized these metrics in traumatic brain injury (K et al., 2020; JM et al., 2015), and more broadly in the fields of fMRI and network neuroscience (M et al., 2017; Kazeminejad et al., 2018; M et al., 2018). There is a wide array of network measures available that can study brain activity at different levels, and it must be emphasized that the results of this study in no way disprove the potential relationship of other network measures and functional outcome in critically ill TBI patients. Nevertheless, we reasoned that a large-scale testing of different network measures would inevitably amount to an increased risk of false results, justifying our decision to limit the number of comparisons to only a few metrics.

Publication bias entails that studies with null or non-significant results, such as the present study, are considerably less likely to be published (Song et al., 2010; Easterbrook et al., 1991). However, we believe that it is important to share evidence which disproves widely believed theories. The results of this study disproved our main hypothesis, suggesting that rs-fMRI brain network connectivity patterns may not be related to long-term functional outcomes following TBI in need of NCCU treatment. These results question the prognostic utility of global network biomarkers derived using rs-fMRI in critically ill TBI patients. Therefore, we believe that it is of great importance that our results are disclosed to the scientific community, even though they were null findings.

## 5 Conclusion

Our findings suggest that that PC and MDZ values are not significantly associated with functional outcome in critically ill TBI patients.

## Disclosures

EPT acknowledges funding support from the Swedish Society for Medical Research, StratNeuro Start-up Grant (Karolinska Institutet), Region Stockholm (Clinical Research Appointment) and the Swedish Brain Foundation (FO2019-0006).

PF was supported by the Swedish Research Council (grant no. 2020-03288) and the Swedish e-Science Research Center.

## Declaration of Competing Interest

The authors declare that they have no known competing financial interests or personal relationships that could have appeared to influence the work reported in this paper.
