# Supplementary material for: Meso-scale network analysis of resting state-fMRI brain network connectivity performs poorly as a prognostic tool in critically ill traumatic brain injury patients
Source: Neuroimage Rep. 2022 Jan 10;2(1):100079. doi: 10.1016/j.ynirp.2022.100079 (PMC12172832; doi:10.1016/j.ynirp.2022.100079)
Supplement: Multimedia component 1 [file mmc1.docx]

**Supplemental Figure 1.**


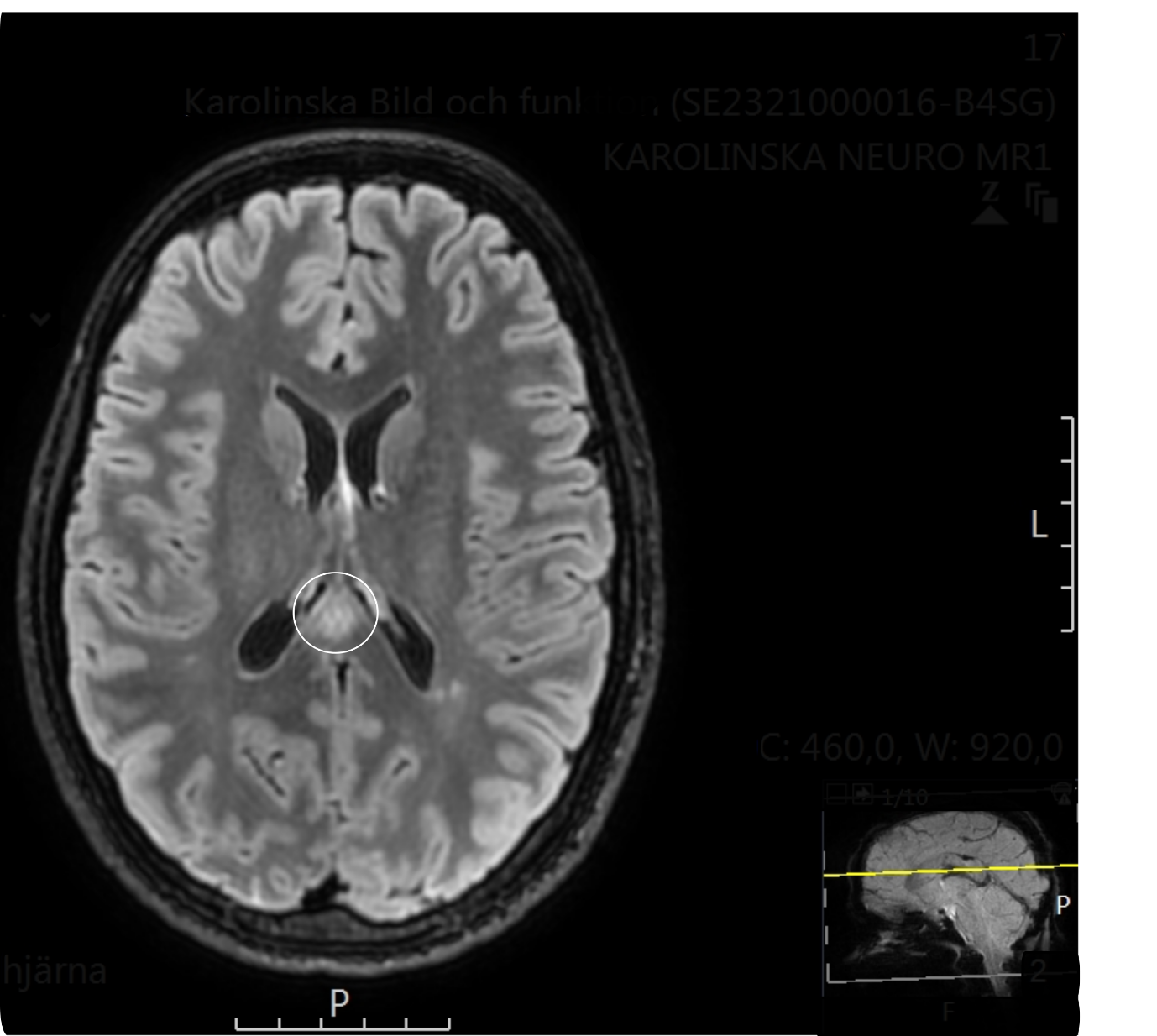


Traumatic axonal injuriy detected using the Fluid attenuated inversion recovery (FLAIR) magnetic resonance imaging (MRI) pulse sequence in the splenium of the corpus callosum, marked with a white circle.

**Supplemental Figure 2.**


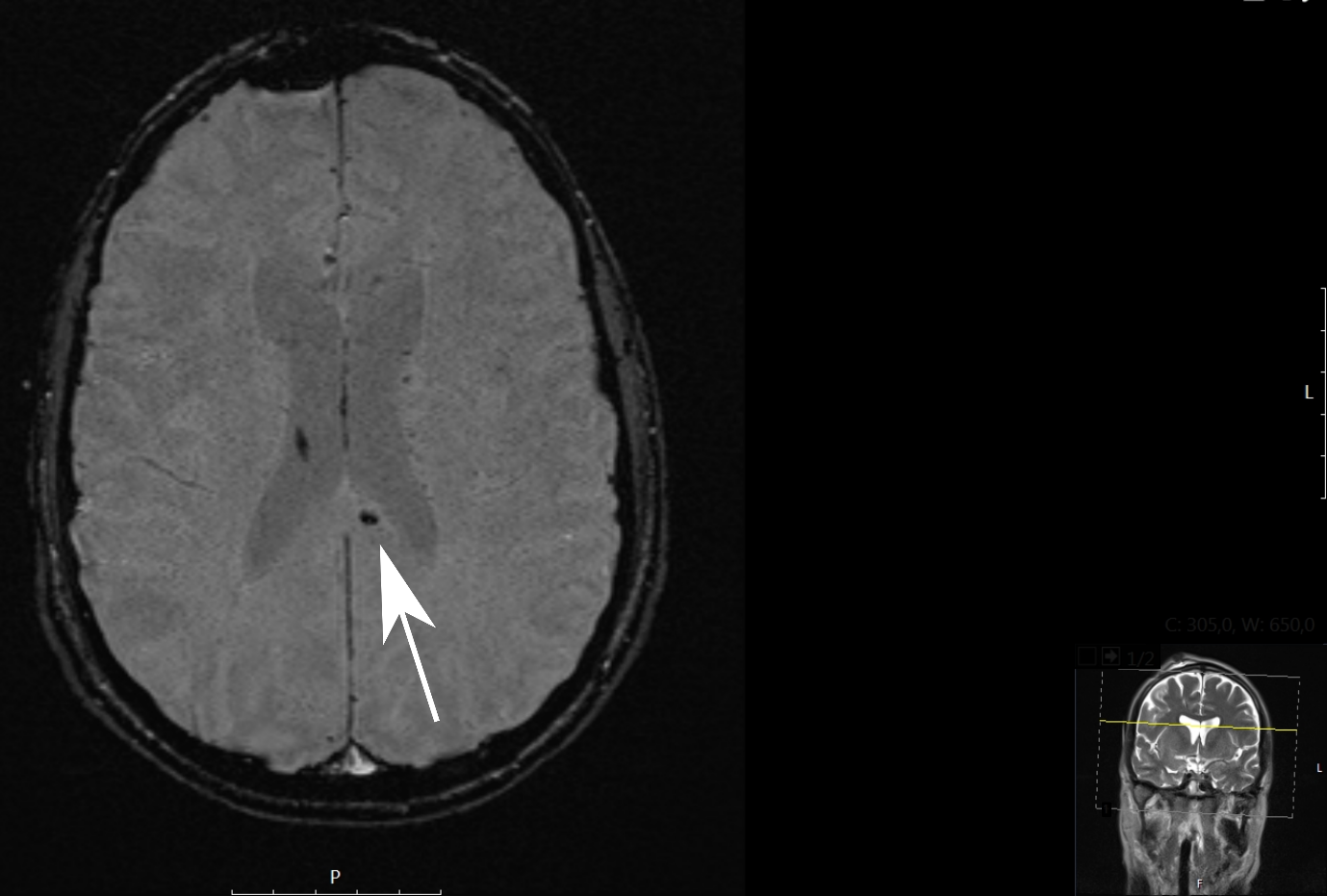


Traumatic axonal injuriy detected using the Susceptibility-weighted imaging (SWI) magnetic resonance imaging (MRI) pulse sequence in the splenium of the corpus callosum, marked with a white arrow.

**Supplemental Figure 3.**


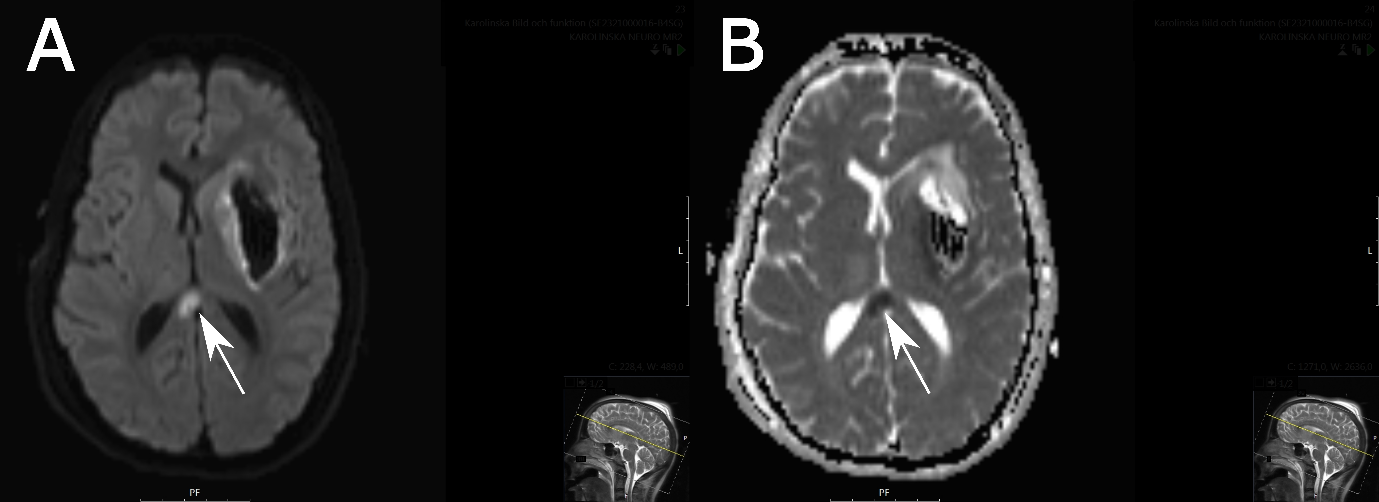


Traumatic axonal injuries exhibiting a pattern of restricted diffusion in the splenium of the corpus callosum, identified by a characteristic hyperintensity on the isotropic diffusion map (A) and a concurrent hypointensity on the Apparent diffusion coefficient (ADC)-map (B).

**Supplemental table 1.** Overall performance of prediction models when including the proportion of excluded voxels among the nuisance regressors.

| **Predictor** | **Pseudo-R^2^** | **AUC** | **AIC** |
| --- | --- | --- | --- |
| Nuisance regressors | 0.38 | 0.80 | 60.3 |
| DMN + Nuisance regressors | 0.39 | 0.81 | 61.6 |
| Somatomotor + Nuisance regressors | 0.38 | 0.80 | 62.3 |
| Visual + Nuisance regressors | 0.38 | 0.80 | 62.2 |
| SalVentAttn + Nuisance regressors | 0.49 | 0.86 | 58.7 |
| DorsAttn + Nuisance regressors | 0.39 | 0.82 | 61.5 |
| Control + Nuisance regressors | 0.47 | 0.83 | 60.0 |
| Limbic + Nuisance regressors | 0.42 | 0.83 | 62.1 |
| AMI + Nuisance regressors | 0.49 | 0.81 | 56.9 |

**Supplemental table 2.** Coefficients and p-values for each of the investigated connectivity measures when including the proportion of excluded voxels among the nuisance regressors.

| Model | Predictor | Coefficient | p value | adj p value |
| --- | --- | --- | --- | --- |
| SalVentAttn + Nuisance regressors | Median MDZ | -11.30 | 0.05 | 0.31 |
| Control + Nuisance regressors | Median MDZ | -6.20 | 0.06 | 0.31 |
| Limbic + Nuisance regressors | Median PC | 44.69 | 0.24 | 0.69 |
| SalVentAttn + Nuisance regressors | Median PC | 21.64 | 0.37 | 0.69 |
| DorsAttn + Nuisance regressors | Median PC | 21.08 | 0.38 | 0.69 |
| DMN + Nuisance regressors | Median PC | 8.67 | 0.41 | 0.69 |
| Limbic + Nuisance regressors | Median MDZ | 1.42 | 0.55 | 0.79 |
| Visual + Nuisance regressors | Median PC | -4.80 | 0.75 | 0.93 |
| AMI + Nuisance regressors | AMI | -0.94 | 0.83 | 0.83 |
| Control + Nuisance regressors | Median PC | 1.39 | 0.94 | 0.99 |
| Somatomotor + Nuisance regressors | Median PC | 0.06 | 0.99 | 0.99 |
